# New Regression Models to Evaluate the Relationship between Biomechanics of Gymnastic Vault and Initial Vault Difficulty Values

**DOI:** 10.2478/v10078-012-0085-6

**Published:** 2012-12-30

**Authors:** Almir Atiković

**Affiliations:** 1 University of Tuzla, Faculty of Physical Education and Sport, Bosnia and Herzegovina.

**Keywords:** Modeling, Code of Points, Gymnastics, Biomechanics

## Abstract

The main objective of this paper was to determine the relationship between biomechanical parameters of vault flights with respect to new models of initial vault difficulty values in men’s artistic gymnastic. The study sample included vaults (n=64) and models (n=5) from the 2009 Code of Points (CoP) of the Federation International of Gymnastics (FIG). The dependent variable included all difficulty values ranging from 2–7.2 points, while the sample of independent variables included twelve biomechanical parameters. After implementing the regression analysis, it could be established that the best model derived only the second flight phase with 95% of explained variance.

## Introduction

Uniform instructions on the Code of Points (CoP) in gymnastics under the Federation International of Gymnastics (FIG) date back to 1949. Every four years after the Olympic Games, the FIG Technical Committee improves and further develops the CoP. Biomechanics research in gymnastics is a growing area of interest, especially when related to scoring of vault difficulty. Physical parameters of vaults are generally-known (Brueggeman, 1994; Prassas, 1995; 2006; Krug, 1997; Takei, 1991; 1998; 2007; Takei et al., 2000; Čuk and Karácsony, 2004; Naundorf et al., 2008; Marinšek, 2010; Ferkolj, 2010).

**Figure 1:**
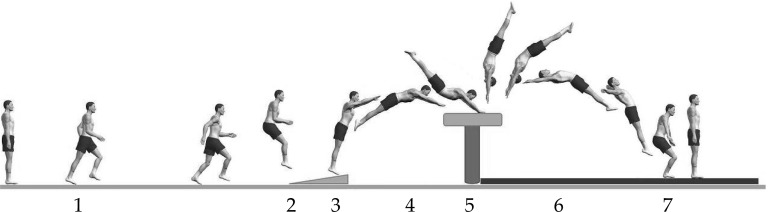
Vault seven phases (Prassas, 2002; 2006; Čuk, Karácsony, 2004; Takei, 2007; Ferkolj, 2010, Atiković and Smajlović, 2011). Vault phases: 1-run, 2-jump on springboard, 3-springboard support phase, 4-first flight phase, 5-support on the table, 6-second flight phase, 7-landing.

**Figure 2:**
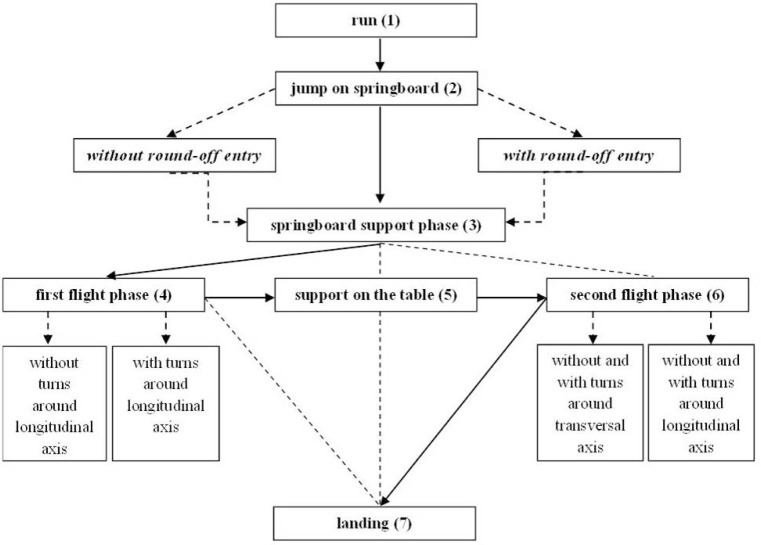
Schematic presentation of a possible jump to the vault (Prassas, 2002; 2006; Čuk, Karácsony, 2004; Takei, 2007; Ferkolj, 2010, Atiković and Smajlović, 2011)

As is shown in Figures 1 and 2, each vault in the CoP can be divided into seven phases: (1) running, (2) jumping on springboard, (3) springboard support, (4) first flight phase (1^st^ fp), (5) table support, (6) second flight phase (2^nd^fp), and (7) landing (Prassas, 2002; 2006; Čuk and Karácsony, 2004; Takei, 2007; Ferkolj, 2010, Atiković and Smajlović, 2011). However, CoP have not always followed the ever-changing nature of vaults. More specifically, rules have been late when it comes to the definition of the vault difficulty level. Based on the 2009 CoP with inclusion of the saltos in the 2^nd^fp, the vault difficulty becomes defined primarily by body position (tucked, piked or stretched) and the number of rotations around the transversal and longitudinal body axis in the 1^st^ fp and 2^nd^fp.

Over the years, difficulty values (DV) have changed on the basis of the total number of rotations performed around transversal and longitudinal axes in the 1^st^fp and 2^nd^fp. Usually the CoP rewarded each new vault with more DV, and old vaults had to be awarded fewer DV although the vault remained the same.

Takei and colleagues (1991, 1998, 2000, 2007) thoroughly dealt with this problem and made a significant contribution in the analysis and modelling of the vault phases for both female and male gymnasts. This work identified mechanical variables that govern the successful performance of a vault. Prassas (2002) schematically presented what factors contribute to a successful vault; some factors are independent while others are under the control of the gymnast. The present work begins with an attempt to identify important variables contributing to successful performance. Generally, the gymnast builds up kinetic energy during a sprint and that energy is partitioned into linear and angular momenta during the springboard phase. These momenta within the constrains of maintaining form requirements and set springboard vaulting table distance dictate the linear and angular momentum carried into the vaulting table. During vaulting table contact, the gymnast interacts with the vaulting table to further refine the post flight linear and angular momentum requirements to achieve the vault’s desired distance, height and rotations. To enable the gymnast to land safely and without additional steps or a fall, landing with the correct body angle increases the chances of “sticking“ the landing by enabling the gymnast to successfully utilize the ground reaction forces to stop rotation and therefore, rely less on musculature. Schwiezer (2003) determined mechanical variables important for optimal vault performance: variation of the positions of the hands, forces of reaction during the support phase of the hands, minimal distance between body center of gravity (BCG) and the far edge of the table while crossing the table, minimal and maxium distances between certain parts of the body and the far edge of the table while crossing the apparatus, position at which the gymnast hits the vaulting board, distance of the vaulting board, and landing distance behind the table.

Čuk and Karácsony (2004) presented biomecahnical characteristics of the vault and the most important factors for a successful vault jump. These factors included mophologic characteristics, run velocity, length of flight on the springboard, duration of board contact, position of feet from springboard edge, duration of 1^st^fp, duration of support on table phase, duration of 2^nd^fp, height of jump, moment of inertia (*J*) in x and y axis, distance from take-off 2^nd^fp, and landing. Čuk et al. (2007) researched the relationship between the difficulty values (DV) of vault and runway velocity in top level male artistic gymnasts. The correlation between each gymnast’s runway velocity and DV of the vault was much lower than the correlation between average velocity of the jump and its DV.

Furthermore, there are many studies reporting of successful valut preformance such as vault run speed, maximum speed on springboard, 1^st^fp and 2^nd^fp (Sands and McNeal, 1995; Krug et al., 1998; Schwiezer, 2003; Čuk and Karácsony, 2004; Takei, 2007; Čuk et al., 2007; Naundorf et al., 2008; Veličković et al., 2011, Brehmen and Naudorf, 2011, Atiković and Smajlović, 2011).

According to the CoP, biomechanical characteristics are important criteria for calculation of DV values. Therefore, it is important to evaluate the DV values from the perspective of biomechanics. The aim of this paper was to determine which biomechanical parameters of five models define the initial vault DV.

## Material and Methods

### Participants

The study of biomechanical samples included 64 of 115 different gymnastics vaults as listed in the 2009 CoP, from which we extracted twelve independent variables. The sample of dependent variables included difficulty values CoP ranging from 2 to 7.2 points.

The paper presents five new models of forming the initial vault difficulty values. During the data collection, we were unable to reach all vaults since some of them had not been performed in the last 20 years (e.g. second group vaults). We analyzed all materials and video recordings from large world men’s competitions of rougly 30 different vaults, accounting for one quarter of all vaults. Some durations parameters: vault run speeds – maximum speed on springboard, 1^st^fp and 2^nd^fp and duration of support on table phase determined as the average value from all vaults were calculated from elite gymnasts (*n*=230) performing at the 2006 World Chapionship in Aarhus (Denmark).

### Procedures

Kinematic analysis was performed using an APAS - Ariel Performance Analysis System (Ariel Dynamics Inc., SanDiego, Ca) in conjunction with the Sušanka et al. (1987) fifteen-segment body model defined with 17 points. All jumps were recorded during the competition using two orthogonal SVHS cameras at 50 frames per second. BCG velocity on springboad, duration of the 1^st^fp and 2^nd^fp and duration of support on table phase were obtained from our research.

Body postures and *J* in vault phases are taken as model values. Average body positions and medium values, based on former research were considered in the support phase at group vaults. To simplify the model, only medium value for an individual group of vaults was taken, because vaults can be performed in different positions (e.g. handspring forward and salto forward) and can be performed either with the presented position in support on the table or with the higher position in the moment of support on the table.

The sample of independent variables consists of degrees of turns in the x axis (α_x_) and y axis (α_y_) for the 1^st^fp and 2^nd^fp based upon the CoP (FIG, 2009) and defined by the quantity of rotations in degrees. The moment of inertia (*J*) was calculated by a cylindric model of Petrov and Gagin (1974), (*J=ml^−1^/12*) for the 1^st^fp and 2^nd^fp and the J in the x and y axes. In this model, *l* is the mean distance beetwen the lowest and highest points of the body for the x axis or the distance between the left-most and right-most points of the body for the y axis. The morphologic data of vault specialists i.e. body height (1.673 m) and body mass (68.15 kg), were taken from Čuk and Karácsony (2004), within the Dempster body model by Winter (1979) and g = 9.81 m/s^−1^.

### Analysis

In analyzing descriptive parameters of variables applied in vaults, the Kolmogorov-Smirnov (KS) test was applied to determine the normality of distribution of the results for further multivariate analysis. Factor analysis was used to determine the latent structure of a set of variables applied using the method of principal components. Furthermore, in order to identify the number of significant principal components, Varimax-Kaiser Normalization was employed. As a result, we present new DV prediction models which are defined on the basis of important biomechanical parameters and individual phases of the vault flight. Five models are offered for the formation of the initial rating, where in three models, the values given are multiplied by the biomechanical parameters. We took into consideration correlations and multiple correlations at the significance level of *p* < 0.05. The models for the analysis of biomechanical variables are as follows:

***Model A****the sum of all values of the entire flight:*
  1. *(BCG velocity on springboard (m/s) + alpha in x axis 1^st^fp (°) + alpha in y axis 1^st^fp (°) + time of 1^st^fp (s) + Jx axis 1^st^fp (kg*·*ms*^−1^) + Jy axis 1^st^fp (kg*·*ms^−1^) + time of support on the table (s) + alpha in x axis 2^nd^fp (°) + alpha in y axis 2^nd^fp (°)****+****time of 2^nd^fp (s) + Jx axis 2^nd^fp (kg*·*ms^−1^) + Jy axis 2^nd^fp (kg*·ms^−1^))*
***Model B****the sum of all values 2^nd^fp:*
  1. *(alpha in x axis 2^nd^fp (°) + alpha in y axis 2^nd^fp (°)****+****time of 2^nd^fp (s) + Jx axis 2^nd^fp (kg*·*ms^−1^) + Jy axis 2^nd^fp (kg*·*ms^−1^))*
***Model C****with emphasis on 1^st^fp Z values:*
  1. (BCG velocity on springboard (m/s) * 0.4 + alpha in x axis 1^st^fp (°) * 0.15 + alpha in y axis 1^st^fp (°) * 0.15 + Jx axis 1^st^fp (kg·ms^−1^) * 0.15 + Jy axis 1^st^fp (kg·ms^−1^) * 0.15)
***Model D*** *with emphasis on 2^nd^fp Z values:*
  1. (alpha in x axis 2^nd^fp (°) * 0.25 + alpha in y axis 2^nd^fp (°) * 0 .25***+***time of 2^nd^fp (s) * 0.3 + Jx axis 2^nd^fp (kg·ms^−1^) * 0.1 + Jy axis 2^nd^fp (kg·ms^−1^) * 0.1)
***Model E*** *with emphasis on understanding the significant weights as described by experts in the Z value researches conducted so far:*
  (BCG velocity on springboard (m/s) * 0.30 + alpha in x axis 1^st^fp (°) * 0.025 + alpha in y axis 1^st^fp (°) * 0.025 + time of 1^st^fp (s) * 0.1 + Jx axis 1^st^fp (kg·ms^−1^) * 0.025 + Jy axis 1^st^fp (kg·ms^−1^) * 0.025 + time of support on the table (s) * 0.1 + alpha in x axis 2^nd^fp (°) * 0.1 + alpha in y axis 2^nd^fp (°) * 0.1***+***time of 2^nd^fp (s) * 0.15 + Jx axis 2^nd^fp (kg·ms^−1^) * 0.025 + Jy axis 2^nd^fp (kg·ms^−1^) * 0.025)

## Results

The analysis of results vault values with twelve biomechanical parameters and five models in the men’s artistic gymnastics begins with vaults (*n*=64), models (*n*=5), *J* for various body positions in the 1^st^fp and 2^nd^fp. It results with a matrix of characteristic roots and explains parts of common variance, structure matrix and regresive analysis in latent space.

Table 1 shows the results of the KS test of normality of distribution. Four variables do not satisfy the normal distribution: time of 2^nd^fp (KS test: .697; *p* < 0.747), CoP – FIG, 2009 (KS test: .758; *p* < 0.614), BCG velocity on springboard (KS test: 1.018; *p* < 0.252) and time of support on the table (KS test: 1.203; *p* < 0.111). Despite the resulting distribution of these variables, if the analyses included all existing flights, including some very light flights, the results could have been different than obtained.

Moment of inertia was calculated for each model (*J/g*) for various body positions in the 1^st^fp and 2^nd^fp (Atiković and Smajlović, 2011) e.g. 1^st^ group (1.706 *kg*·*ms^−1^*), 2^nd^ (1.978 *kg*·*ms^−1^*), 3^rd^ (1.771 *kg*·*ms^−1^*), 4^th^ (1.874 *kg*·*ms^−1^*), 5^th^ (1.145 *kg*·*ms^−1^*), tucked (0.458 *kg*·*ms^−1^*), piked (0.738 *kg*·*ms^−1^*), stretched (0.127 *kg*·*ms^−1^*), arch in 4^th^ group (0.555 *kg*·*ms^−1^*).

Given that the results of all models are expressed in different units of measurement, and in order to perform comparison of those measured, it was necessary to transform the results into the original Z value. When we look at the range of results (Table 1) in the CoP model (FIG, 2009), it amounts to 5.2 from minimally to maximally difficult flight. For model A (and B) there were three (two) times higher coefficient of discrimination than in CoP. Compared to the existing CoP (FIG, 2009), discrimination reduced by 50% was found in models C, D and F.

The predictor system (Table 2) of all twelve biomechanical variables in model B (R^2^) explains 95% of the common variables with the criteria. The coeficient of multiple correlation of the entire predictor system of variables with criteria model B amounts to (R) 0.97. Model B proved to be the best model significantly differentiating the differences between the current DV and score all four factors (Table 3).

We analyzed the impact of the individual variables (Table 3) and showed that the highest and statistically most important influence of the criteria variables are as follows: *REGR 3 - factor degrees turns around longitudinal axis* (*β*: 0.933, *p* < 0.001), *REGR 1 - factor turns around transversal axis in 2^nd^fp* (*β*: 0.167, *p* < 0.001), *REGR 2 - factor 1^st^fp* (*β*: 0.065, *p* < 0.001) and the *REGR 4 - factor support on the table* (*β*: −0.227; *p* < 0.001). The statistical significance of the predication model means that the present vault difficulties, based on teh CoP, were defined by the four variables of the vault phases. The regressive analysis clearly shows that the initial value prediction is very high.

In model B factor support on the table (*β*: −0.227; *p* < 0.001) has a negative mark, suggesting that in order for the gymnast to successfully perform 2^nd^fp and increase the amount of rotation around the longitudinal axis of the body, he should spend less time in this flight phase. Analysing all models we can conclude that the B model proved to be the best model that shows significant difference in comparison with the existing final score. It also showed that all four factors were statistically significant.

Upon completing the matrix structure, we found four signficant variables important to vault flight. The first major component of this study consists of turns around transversal axis in 2^nd^fp. The most significant projections (correlations) of vectors of manifest variables on the first principal component have the variables: alpha 2^nd^fp in x axis (°) (.951), time of 2^nd^fp (s) (.838), BCG velocity on springboard (m/s) (.796). Some authors have obtained similar results (Bruggemann, 1994; Prassas, 2002; Schwiezer, 2003; Čuk, 2004; King and Yeadon, 2005; Takei, 2007). The highest projections of the first principal component show variables defining the amount of rotation in 2^nd^fp around transversal axis and temporal parameters. It can be concluded that the first principal component with 26.4% explained variance is turns around transversal axis in 2^nd^fp.

The second principal component has the highest projections on the following variables: Jy axis in 1^st^fp (*kg*·*ms^−1^*) (.894), Jx axis in 1^st^fp (*kg*·*ms^−1^*) (.725), alpha 1^st^fp in x axis (°) (−.828) characterized by the body position during the flight phase in y and x axis. It can be concluded that the second major component with 23.4% explained variance is J and 1^st^fp. The higher the number of turns around the transversal axis is, the smaller is the number of turns around longitudinal axis. Additionally, this parameter is defined by parameter J (Takei et al., 2000).

The most significant projections to the third major component have variables: alpha in 2^nd^fp y axis (°) (.859), Jy axis 2^nd^fp (*kg*·*ms^−1^*) (.848), defined as a factor of turns around longitudinal axis. Third major component with 13.3% explained variance consists of degrees turns around longitudinal axis. Bruggemann (1987) and Kwon (1996) noted that the DV is often increased by adding sea rotations of somersaults into its basic form.

The most significant projection to the fourth major component is the variable of table support, which can be conditionally defined as a factor. The fourth major component is defined by 9.1% explained variance. Takei et al. (2000) used correlation analysis to establish the strength of the relationship between the causal mechanical variables identified in the model and the judges’ scores. Gymnasts have a very short period of time to prepare for the continuation of the vault. In a study of handspring double salto forward tucks, Takei (2007) analyzed strength of the relationship between the mechanical variables identified and the judges’ scores. Significant correlations indicated that the higher judges’ scores were related to five positive mechanical variables and positively related to seventeen variables in the deterministic model.

## Discussion and Conclusions

This paper presented five new models of the formation of the initial vault difficulty values. Deterministic models of vault flight have been mainly determined from a single flight (Kerwin et al., 1993; Grervais, 1994; Yeadon et al., 1998; Takei, 2007). For model A (which summed the Z values for an entire flight) and model B (which summed all Z values for the 2^nd^fp) there were three and two times higher coefficient of discrimination than in the CoP, respectively. For the remaining models were found 50% smaller discrimination than the existing CoP. The impact of individual variables on the criteria variable CoP and models from A to F lead us to the conclusion that members of the Men’s Technical Committee FIG had in mind a simple model of the CoP easily determining the vault difficulty level. The present vault DV model of the CoP is not too complicated. However, it clearly does not differentiate from the difficulty vault groups and most important biomechanical components.

The results of this study in model B can be used for coaching team competition, all around, individual events, as well as the authors of next CoP for the next Olympic cycle. Bearing in mind the results, one could make a better model of determining the DV of a vault. With the regression analysis of selected models, the information presented within the five models gives us a model B on the impact of variables applied to the prediction success of the initial rating of the treated criterion variable. Prediction of system variables explained (R^2^) 95% of the common variability with the criterion, while the connectivity of the entire prediction system of variables with the criterion, the coefficient of multiple correlation is (R) 0.97.

The analysis of the individual variables impact showed that the factor degree turns around longitudinal axis (*β*: 0.933, *p* < 0.001) is statistically the most significant impact on the criterion variable. Partial regression coefficients suggest that the prediction of the impact of predictors on the criterion variable can be made using all four parameters of the predictor variables. A factor with a negative indication is the support on the table *(β*: −0.227, *p* < 0.001), suggesting that the gymnasts need to spend as little time in the phase of flight as possible in order to successfully execute the second phase of the flight and increase the amount of rotation around the longitudinal axis of the body. With this type of research we confirmed that the initial ratings in the vault from a biomechanical point of view can be more objectively determined by the expert of the Men’s FIG Technical Committee.

## Figures and Tables

**Table 1:** Descriptive characteristics and the Kolmogorov - Smirnov test normality of the distribution for vaults (n=64) and models (n=5)

Valid (*n*=64) vaults	R	Min	Max	M	SD	Skew.	Kurt.	KS test	*p*
CoP – FIG, 2009 (points)	5.20	2.00	7.20	5.021	1.366	−.174	−.617	.758	.614
Model A	14.745	−8.495	6.250	.000	3.060	−.263	.032	.487	.972
Model B	10.177	−5.722	4.455	.000	2.127	−.171	−.071	.377	.999
Model C	2.088	−.811	1.276	.000	.473	.643	.566	.865	.442
Model D	2.502	−1.435	1.067	.000	.567	−.334	−.202	.563	.909
Model E	2.539	−1.112	1.427	.000	.527	.592	.778	.888	.410

n – no. of performances; R - range; Min, Max – lowest and highest value; M – mean; SD – standard deviation; Skew., Kurt. – coefficients of skewness and kurtosis; KS test – Kolmogorov Smirnov test normality of the distribution - significant at the (p < 0.05) level.

**Table 2:** The regressive analysis of the criteria variable CoP (FIG, 2009) and models from A to E

Models	CoP (FIG, 2009)	Model A	Model B	Model C	Model D	Model E
R	.842^(a)^	.799^(a)^	.977^(a)^	.792^(a)^	.888^(a)^	.888^(a)^
R^2^	.709	.638	.955	.628	.788	.788
Adjusted R^2^	.689	.614	.952	.603	.774	774
Std. E. of the Estimate	.762	1.901	.468	.298	43.161	17.217
ΔR^2^	.709	.638	.955	.628	.788	788
ΔF	35.948	26.036	310.568	24.896	54.809	54.920
df1	4	4	4	4	4	4
df2	59	59	59	59	59	59
p	.000	.000	.000	.000	.000	.000

a. Predictors: (Constant), REGR 1 - factor turns around transversal axis in 2^nd^f, REGR 2 - factor 1^st^fp, REGR 3 - factor degrees turns around longitudinal axis, REGR 4 - factor support on the table. Dependent Variable: Code of Points – CoP (FIG, 2009), Model A - the sum of all Z values of the entire flight, Model B - the sum of all Z values 2^nd^fp, Model C - with emphasis on 1^st^fp Z values, Model D - with emphasis on 2^nd^fp Z values, Model E - with emphasis on understanding the significant weights as described by experts in the Z.

**Table 3:** The impact of individual variables on the criteria variable CoP (FIG, 2009) and models from A to E

Models	CoP (FIG, 2009)		Model A		Model B		Model C		Model D		Model E	

	*β*	*p*	*β*	*p*	*β*	*p*	*β*	*p*	*β*	*p*	*β*	*p*
REGR 1	.757	.000	.345	.000	.167	.000	.657	.000	.600	.000	.606	.000
REGR 2	−.127	.075	.227	.005	.065	.023	.148	.068	.090	.140	.078	.197
REGR 3	.316	.000	.653	.000	.933	.000	.089	.269	.611	.000	.613	.000
REGR 4	−.140	.051	.202	.012	−.227	.000	.408	.000	−.216	.001	−.197	.002

Predictors: (Constant), REGR 1 - factor turns around transversal axis in 2^nd^f, REGR 2 - factor 1^st^fp, REGR 3 - factor degrees turns around longitudinal axis, REGR 4 - factor support on the table. Dependent Variable: Code of Points – CoP (FIG, 2009), Model A - the sum of all Z values of the entire flight, Model B - the sum of all Z values 2^nd^fp, Model C - with emphasis on 1^st^fp Z values, Model D - with emphasis on 2^nd^fp Z values, Model E - with emphasis on understanding the significant weights as described by experts in the Z value researches conducted so far.
